# Liliana Lubińska (1904–1990)

**DOI:** 10.1007/s00415-020-09787-3

**Published:** 2020-03-16

**Authors:** Karol Wiśniewski, Joanna Wojtkiewicz

**Affiliations:** Department of Pathophysiology, School of Medicine, University of Warmia and Mazury, Olsztyn, Poland

**Fig. 1:**
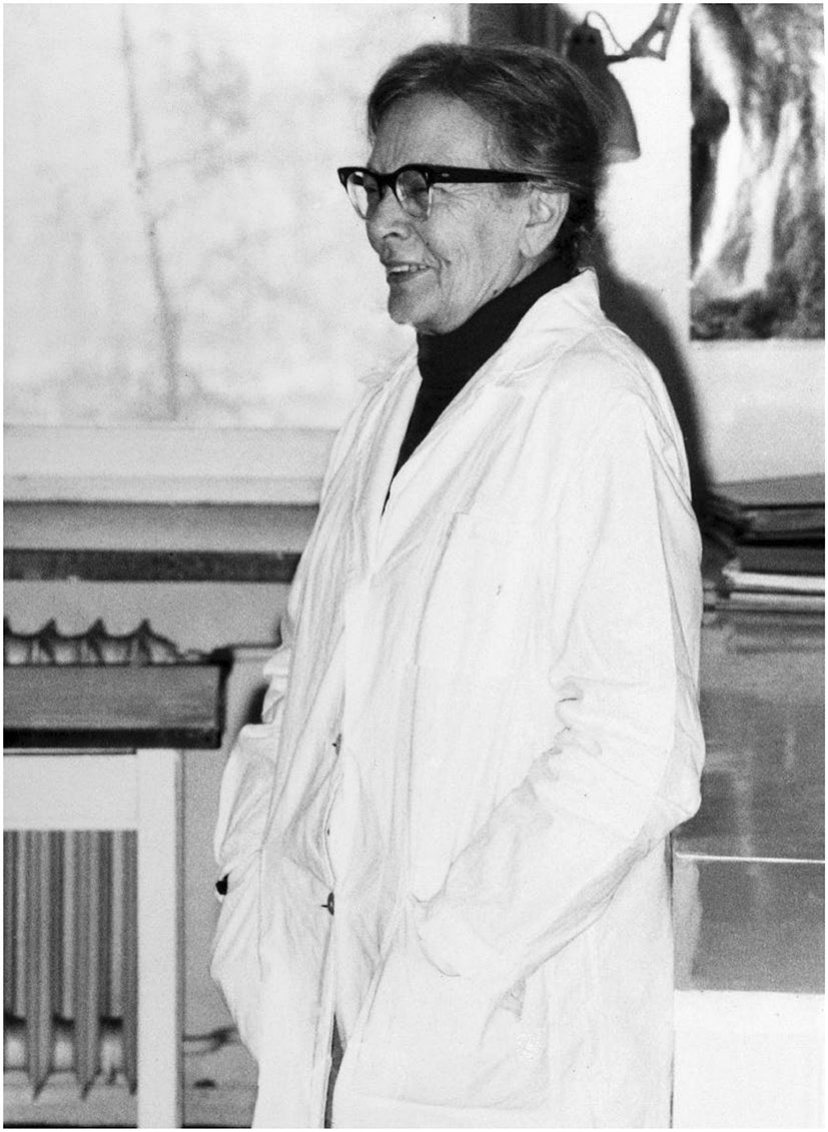
Liliana Lubińska. Photo from www.ptbun.org.pl/

Liliana Lubińska (Fig. 1) was born on 14 October 1904 in Łódz. She was a famous Polish neuroscientist who was associated for most of her career with the Nenecki Institute in Warsaw. Lubińska devoted almost the whole of her life to studies of the peripheral nervous system, especially the subject of nerve regeneration and axoplasmic flow [1].

In 1919, she started her education in Secondary School in Warsaw. In 1923, she entered the University of Warsaw to study biology, but after just 1 year, she decided to move to Paris where she began studies in biological sciences at the Sorbonne. In 1927, she obtained her first degree in biological chemistry and physiology. At the same time, she started working in the Physiology department which was headed by Professor Luise Lapicque. 5 years later, she obtained the degree of Doctor of Natural Sciences with honours [1]. For her doctoral work, she was also awarded by the French Academy [1, 2]. In those years, she published eight articles exploring the field of the aforementioned subjects.

Despite her scientific success in Paris, she decided to return to Poland and to obtain the position of an assistant at the Nenecki Institute. Before the outbreak of the Second World War, her scientific work concentrated on the effects of different agents on the excitability of the neuromuscular preparation. Together with Jerzy Konorski and Stefan Miller, she was involved in studying conditional reflexes [1].

The years of the Second World War were not easy for Lubińska. During the siege of Warsaw, she and her husband, Jerzy Konorski, had to leave their house and move to their friends due to bombing of the city [1, 2]. Because they did not want to stay under Nazi occupation, they decided to leave Warsaw as soon as possible. Thanks to the help of their friends, they had the opportunity to work in the Department of Physiology of the Institute of Experimental Medicine in Sukchumi (Russia). During that time, Lubińska concentrated on studying regeneration of peripheral nerves and their excitability [1].

In summer 1945, Lubińska returned to Warsaw. Together with Jerzy Konorski, Stella Nimierkowa, Włodzimierz Niemerko, Staniasława Dembowska and Jan Dembowski, she decided to reactivate the Nenecki Institutue, the building of which had been destroyed. Temporarily, the Nenecki Institue was moved to Łódz, where Lubińska continued her work up to 1955, when the Institute was finally rebuilt in Warsaw [1, 2].

Liliana Lubińska’s academic achievements were very impressive. She published over 80 articles in both Polish and international journals [3]. Her work mainly concerned topics of the peripheral nervous system, nerve regeneration, and axoplasmic flow. The best proof of the innovation, thoroughness and path-breaking nature of Lubińska’s studies is the fact that her works were published in such journals as Nature and The Lancet [3–5]. In her 1954 paper “Form of myelinated Nerve Fibres”, Lubińska presented studies of unstained cat peripheral nerve fibres. The experiments additionally confirmed the existence of a proximo-distal flow of axoplasm in the axon [4]. In the 1950s, she also investigated the outflow of axoplasm from cut ends of nerve fibres [6]. Lubińska was also involved in investigation of intercalated internodes in nerve fibres. In her study, she noticed that the nucleus of the Schwann cell is always located in the middle of the short internode. Her results also supported earlier scientific reports and led to better understanding of nerve regeneration [5].

Liliana Lubińska is mostly known for her concept of bidirectional axonal transport. She presented her theory in 1962 [7]. In 1971, with her friend Stella Niemerko, she published one of her most famous and most cited works, entitled “Velocity and intensity of bidirectional migration of acetylcholinesterase in transected nerves”. In it, Lubińska presented the approach of the migration of acetylcholinesterase in transected nerves of dogs. The results showed not only the fact that the transport is bidirectional, but also that the velocity of proximo-distal transport is about twice the velocity of disto-proximal transport (260 mm/day vs 134 mm/day, respectively). The results also pointed out that the intensity of migration is also twice as much in the descending direction as in the opposite one. It is also worth mentioning that the results supported the idea of ‘fast transport’ [8]. In her last work, Lubińska concentrated on the role of axoplasmic flow of trophic factor. She was trying to explain the possible role of trophic factor in the degeneration of phrenic nerves [9].

Liliana Lubińska died at the age of 86 in 1990. She was buried in North municipal cemetery in Warsaw [10]. The character of Liliana was described by her friend, Stella Niemerko, in the following words: “Liliana was a scientist of uncommon intellectual qualities: her invention was joined with profound knowledge of biological phenomena: she was an excellent experimenter and I think that for a young person, the work with her was an extremely useful lesson for the whole life” [1].
